# Three Mental Health Symptoms of Frontline Medical Staff Associated With Occupational Stressors During the COVID-19 Peak Outbreak in China: The Mediation of Perceived Stress and the Moderation of Social Support

**DOI:** 10.3389/fpsyg.2022.888000

**Published:** 2022-05-27

**Authors:** Yang Zou, Yinhuan Lu, Fan Zhou, Xiaoyue Liu, Arlette J. Ngoubene-Atioky, Kewei Xu, Liuzhi Hong, Guanghui Shen, Huifen Wu, Zhaohong Cai, Yanlong Liu, Li Chen, Donger Bao

**Affiliations:** ^1^School of Mental Health, Wenzhou Medical University, Wenzhou, China; ^2^Affiliated Cixi Hospital, Wenzhou Medical University, Ningbo, China; ^3^Department of Pediatrics, The Second Affiliated Hospital and Yuying Children’s Hospital of Wenzhou Medical University, Wenzhou, China; ^4^Center for Psychology, Goucher College, Baltimore, MD, United States; ^5^The Affiliated Xiangshan Hospital of Wenzhou Medical University, Ningbo, China

**Keywords:** COVID-19, frontline medical staff, occupational stressors, perceived stress, social support, anxiety, depression, insomnia

## Abstract

The outbreak of COVID-19 epidemic has increased work demands for medical staff and has a certain impact on their mental health. The present study aimed to examine the role of perceived stress and social support in explaining the association between the occupational stressors and three mental health symptoms (i.e., anxiety, depression, and insomnia) of frontline medical staff. Five hundred twenty five frontline medical staff were investigated online after the outbreak of the COVID-19 (16 February, 2020–2 March, 2020) in China. The results found that the prevalence of anxiety, depression, and insomnia among frontline medical staff were 39.8, 29.9, and 37.9%, respectively. Occupational stressors were associated with anxiety, depression, and insomnia symptoms. Perceived stress significantly mediated this link. Social support moderated the second half of the indirect effect of occupational stressors on anxiety and depression symptoms. Under the epidemic situation of COVID-19, for frontline medical staff, high perceived stress and low social support may increase vulnerability for mental health symptoms triggered by occupational stressors. Thus, improving the social support and promoting the cognitive reappraisal of perceived stress may help to maintain mental health among medical staff.

## Introduction

The World Health Organization (WHO) has declared the outbreak of coronavirus disease 2019 (COVID-19) a pandemic. The COVID-19 virus has spread in more than 220 countries, 5.9 million people have died of COVID-19 and more than 430 million have been infected by the end of 25 February, 2022 (WHO, 2022). In addition to many deleterious physical health impacts, COVID-19 has had dire consequences on the mental health of the world population (Reskati et al., 2021). Existing research has suggested that the psychosocial effects of COVID-19 are, most of the time, greater and more prolonged than its physical symptoms (Allsopp et al., 2019). This is especially evident for COVID-19 frontline medical staff who have worked tirelessly to mitigate its propagation.

Multiple studies have demonstrated that the elevation of work demands and the necessity to engage in greater and highly stressful work responsibilities have significant negative consequences on the mental health of frontline COVID-19 medical staff (Sanghera et al., 2020; Zaka et al., 2020; Bizri et al., 2021). However, several studies have also indicated that higher occupational demands do not always trigger mental health symptoms (Wang and Wang, 2019). Such observation has led to the recognition that the association between occupational stressors and mental health symptoms might be indirect. A dearth of research remains regarding resources that can mitigate the harmful effects of occupational stressors. Thus, the current study seeks to investigate the relationship between occupational stressors and mental health symptoms (i.e., depression, anxiety, and insomnia) of frontline medical staff at the peak of COVID-19 epidemic.

Social support is an essential social factor that can mitigate the negative effects of stress. Social support is associated with higher levels of positive mood, fewer health complaints, lower depressive symptoms, minimal sleep problems, and higher life satisfaction (Chen et al., 2009; Xiao et al., 2020). In this study, we focus specifically on whether social support can buffer the impact of occupational demands or stress on depression, anxiety, and insomnia symptoms respectively.

Therefore, the purpose of this study is to (a) establish associations among occupational stressors, perceived stress and mental health symptoms (i.e., depression, anxiety, and insomnia), (b) understand the underlying mechanisms behind these associations, and (c) demonstrate how social support can mitigate the link between occupational demands or stress and depression, anxiety, and insomnia symptoms respectively.

### Occupational Stressors and Mental Health Symptoms of Medical Staff

Occupational stressors refer to social and physical work-related circumstances that challenge the adaptive capabilities and resources of an employee. Occupational stressors may trigger one’s stress response and cause psychological or physical health problems including negative emotions, sleep disturbances, and even cardiovascular disease, when one’s personal coping resources are inadequate (Suleman et al., 2018). Medical institutions are demanding occupational settings. High time pressure, varying workloads, and exposure to traumatic events may place strain on medical staff (Greenslade et al., 2020). Stressors within the medical institutions contribute to high levels of psychological distress and turnover (Walton et al., 2020). Research has shown that medical staff are usually exposed to more demanding and complex chronic and acute occupational stressors than the general population (Zaka et al., 2020; Galbraith et al., 2021).

Since the outbreak of COVID-19, medical institutions in China and even around the world are facing unprecedented challenges. Frontline medical staff have endured months of unimaginable accumulation of work demands, compounded with their own personal obligations in this unforeseeable global disaster (Kang et al., 2020). Their work-life balance which, in the medical field, pre-pandemic, is well-known to be marginally equitable (Ma et al., 2020), have been disproportionally jeopardized during this COVID-19 era.

Psychological distress is another major adverse consequence of the COVID-19 pandemic among the medical staff. A systemic review of the mental health of 69,499 health professionals worldwide at the onset of the COVID-19 from 436 published articles found about 14–45% experienced depression, 12–36% reported anxiety, 5–33% shared having acute stress symptoms, 7–34% met criteria for post-traumatic stress disorder (PTSD), 34–36% had insomnia, and 3–43% experienced occupational burnout (Sanghera et al., 2020). These studies shed valuable information of mental health problems in the high work environment of frontline medical staff. It also begs into questioning the triggering effect of occupational stressors on the mental health symptoms of medical staff. Furthermore, Moreno Fortes et al. (2020) surveyed 440 employees and found that occupational stressors had a significant reverse correlation with positive mental health. Other researchers also suggested that occupational stressors are a significant predictor of anxiety and depression (Mark and Smith, 2012; Wang et al., 2017). It thus seems reasonable to expect a significant link between occupational stressors and mental health symptoms among frontline medical staff during the COVID-19 pandemic.

**H_1_:** Occupational stressors is positively associated with mental health symptoms including anxiety (1a), depression (1b), and insomnia (1c).

### The Mediating Role of Perceived Stress

Despite the robust evidence that suggests an association between negative mental health symptoms and occupational stressors, there remains minimal understanding of how occupational stressors negatively influences mental health (Quick and Henderson, 2016; Williams, 2018).

The cognitive appraisal model of stress asserts that cognitive appraisal is the factor linking stressors to experienced distress, particularly, to mental and somatic symptoms. When exposed to stressors, an individual subjectively assesses the stressors (primary appraisal) and subsequently determines which coping resources can be applied to the situation (secondary appraisal) (Harvey et al., 2010). If the individual appraises that their resources are sufficient to meet the demands of event, they will feel less strained. If the resources are not judged to be sufficient, stress will consume them (Carpenter, 2016). Therefore, it is not whether stressors lead to ill-health, but whether the individual perceives a situation as a threat or not (Harvey et al., 2010; Balieiro et al., 2011). Thus, the appraisal of the potentially stressful event plays an important role in the negative outcomes (Gustafsson and Skoog, 2012).

Perceived stress refers to the psychological response that an individual has after their cognitive evaluation of a stimulus as a threat. Perceived stress is one form of cognitive appraisal that may explain the link between occupational stress and mental health symptoms (Balieiro et al., 2011). Evidence suggests perceived stress have adverse and pessimistic effects on mental health, as measured by insomnia, depression, anxiety, and lower psychological well-being (Yin et al., 2013). In one study, stress perceptions were found to mediate the relationship between stressors and health-related quality of life (Rusli et al., 2008).

Furthermore, field quasi-experiments (Harvey et al., 2010) demonstrate that subjective appraisals of a situation appear to play an important role in stress responses, which have previously been shown to impair health. In line with this model and its supporting research, we presuppose that perceived stress may be one factor linking the association between occupational stressors and mental health symptoms (i.e., anxiety, depression, and insomnia).

**H_2_:** Perceived stress mediates the association between occupational stressors and mental health symptoms including anxiety (6a), depression (6b), and insomnia (6c).

### The Moderating Role of Social Support

While several studies have shown strong evidences for the (in)direct effect of occupational stressors on mental health symptoms via perceived stress, a few other studies have failed to find support for this pathway, suggesting these associations may be conditional on other factors.

The stress-buffering model of social support provides an important perspective in understanding the potential mechanism between the occupational stressors, perceived stress, and mental health symptoms. The stress-buffering hypothesis argues that the social support protects mental health through the indirect pathway of interacting with the stressors or moderating the effect (Cohen and Wills, 1985). In other words, social support may exert a direct effect by reducing the severity with which stressors are perceived, but it can also act as a buffer against the deleterious effects of stressors perception, reducing the activity of pathways that harm health (Cohen and Wills, 1985; Moseley et al., 2021). Based on this model, social support is a buffering factor in the development of psychopathology and adjustment difficulties following experiences of a stressful event(s) (Praharso et al., 2017). Empirical evidence of buffering effect of social support between stressors or stress and mental health problems has rapidly accumulated in recent years. For example, an early and influential study found that the effect of negative daily events on psychological distress significantly decreased as positive social ties increased, which is in accordance with the stress-buffering hypothesis (Okun et al., 1990). Subsequent research has also supported the stress-buffering hypothesis of social support (Lakey and Cronin, 2008; Raffaelli et al., 2013).

Prior finding suggested that social support is not only related to objective occupational stressors, but also related to individual perceived stress (Kneavel, 2021). Occupational stressors are work-related factors, which are objective factors that cause stress in one’s job (Kshtriya et al., 2020), perceived stress is the psychological response to threatening stimuli in the environment after cognitive evaluation (Li and Lyu, 2020). It is crucial to distinguish the buffering effect of social support on occupational stressors and perceived stress. Although a large number of studies have demonstrated that social support moderated or buffered the relationship between stressful experiences and mental health, few studies have noted the role of social support as potential buffers of the stressors-stress-mental health symptoms relationship simultaneously, especially in frontline medical staff who mainly provide health care during the COVID-19 epidemic. Hence, in light of the theory of buffer theory of social support and previous research, this study examines social support as a critical moderator of the relationship between occupational stressors, perceived stress, and mental health symptoms.

**H_3_:** Social support moderates the association between occupational stressors, perceived stress, and mental health symptoms including anxiety (3a), depression (3b), and insomnia (3c).

### The Present Study

**FIGURE 1:**
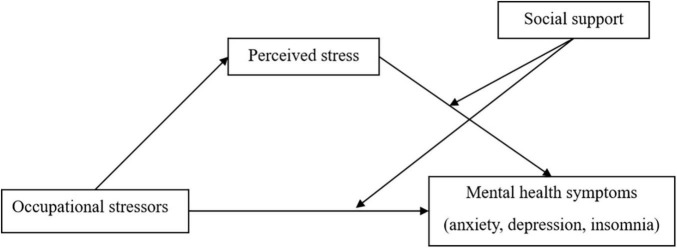
The moderated mediation effect among occupational stressors, mental health symptoms (anxiety, depression, and insomnia), perceived stress, and social support.

In this context, understanding the shifts in the frontline medical staff’ mental health during the COVID-19 pandemic and the factors that may be influencing changes in mental health is relevant to comprehending frontline medical staff’ responses to the ongoing pandemic. Furthermore, few studies have investigated the potential mechanism of occupational stressors and mental health symptoms of frontline medical staff during the COVID-19 pandemic. It is important to understand the effect of perceived stress and social support on this association to effectively prevent and treat psychological distress for frontline medical staff. This may provide some intervention measures to develop public health management in the future. Taken together, the aims of the present study are to (1) estimate the prevalence of and severity of three kinds of mental health symptoms including depression, anxiety, and insomnia among frontline medical staff, (2) identify the mediating role of perceived stress in the link between the occupational stressors and three kinds of mental health symptoms respectively, and (3) evaluate the moderating role of social support in the link among occupational stressors, perceived stress and three kinds of mental health symptoms. The full proposed model is displayed in Figure 1.

## Materials and Methods

### Participants and Procedure

Participants were frontline medical staff residing in Wenzhou, China who directly participated in the fight against COVID-19 by contacting confirmed COVID-19 cases or their specimens in the isolation hospitals (Zhang et al., 2020b). This study recruited participants through purposeful sampling via the website Wen Juan Xing,^1^ a widely used online survey platform in China. According to the data on the 2020 coronavirus cases in China provided by the National Health Commission (NHC), the novel coronavirus outbreak (COVID-19) reached its peak between January 23, 2020 and February 4, 2020 and the number of new cases has steadily declined ever since. By January 29, 2020, 33,000 people had returned to Wenzhou from Wuhan and its neighboring cities, and 172 individuals were confirmed positive cases of COVID-19 in Wenzhou, making the city become the area with the most confirmed cases outside of Hubei Province in China. Lockdown was enacted in Wenzhou to curb the spread of COVID-19 virus on February 5, 2020.

The sample recruitment procedure consisted of a two-step procedure conducted in February 2020. First, a pilot study was conducted with the participation of 10 frontline medical staff from the target population to evaluate clarity, comprehensiveness, and acceptability of questionnaires. Second, in the formal study, links to the survey were sent to frontline medical staff via WeChat (the most widely used social media platform in China). Participants completed and submitted the questionnaire on their smartphones by clicking the survey link. The questionnaires were responded anonymously and voluntarily. An online consent form was provided to the participants before filling out the questionnaire. The survey would not continue unless participants chose the option – “Yes, I consent to take part in this survey.” Hence, a total of 525 frontline medical staff residing in Wenzhou participated. Of the 525 participants, 392 (74.7%) were female, ages ranged from 19 to 60 years-old (*M* = 35.29, *SD* = 7.05 years), 442 (84.2%) at college or higher education levels, and 416 (79.2%) were reported being married. The length of professional medical service ranged from 0 (<1 year) to 44 years (*M* = 12.32, *SD* = 8.66 years). Details of socio-demographic characteristics of the participants are displayed in Table 1. The study was approved by the Ethics Committee of Wenzhou Medical University, and all methods of this study were carried out in accordance with the approved guidelines.

**TABLE 1:** Socio-demographic of the frontline medical staff (*N* = 525).

Variables		*N*	%
Sex	Male	133	25.3%
	Female	392	74.7%
Age: years old	≤30	149	28.4%
	31–50	366	69.7%
	>50	10	1.9%
Education level	High school or below	83	15.8%
	College or above	442	84.2%
Marital status	Married	416	79.2%
	Unmarried	109	20.8%
Length of service: years	≤10	263	50.1%
	10–20	175	33.3%
	>20	87	16.6%

### Measurements

#### Socio-Demographic Data

These data include sex, age, education level, marital status, and length of service.

#### Perceived Stress

The 10-item Perceived Stress Scale (PSS-10) (Leung et al., 2010) was utilized to evaluate perceived stress in clinical settings. Participants responded to each question on a 5-point Likert scale ranging from 0 (never) to 4 (very often), indicating how often they have felt or thought a certain way within the past month. Higher score means of greater perceived stress. Previous studies have shown that the Chinese version of the PSS-10 has good reliability and validity (Wang et al., 2011), and it has demonstrated excellent internal consistency (α = 0.91) in the present study.

#### Social Support

Five items assessing participants’ perceptions of support from (1) family, (2) friends, (3) supervisors, (4) colleagues, and (5) Mental health worker during the COVID-19 epidemic were used as indicators of social support. Responses were recorded on a 4-point scale ranging from “not at all” to “extremely strong.” Example items include “I get help and support I need from my family” and “I get help and support I need from my friend.” A higher mean score indicates stronger social support. The items demonstrated good internal consistency in this sample (α = 0.86).

#### Occupational Stressors

The self-designed questions were used to measure the occupational stressors of frontline medical staff during the COVID-19 outbreak. It consists of three items: (1) work difficulty, (2) work stress, and (3) work risk during the COVID-19 pandemic. Example items include “My work was very difficult during the epidemic” and “My work was at great risk during the epidemic.” Participants were asked to score each item on a 5-point scale, from 1 (Strongly Disagree) to 5 (Strongly Agree). The higher scores the score is, the more occupational stressors. In this study, the items had acceptable an internal reliability (α = 0.77).

#### Self-Reported Symptoms of Mental Health

##### Anxiety

The Generalized Anxiety Disorder Scale-7 item (GAD-7) (Spitzer et al., 2006) was used. Each of the items on the GAD-7 is rated on a 4-point scale from 0 (not at all) to 3 (almost every day). The GAD-7 has been found to be a well-validated screening instrument (Abe et al., 2020); in this study, it had excellent internal consistency (α = 0.94).

##### Depression

The level of depression in participants was assessed using the Patient Health Questionnaire-9 item (PHQ-9) (Kroenke et al., 2001). A 4-point Likert scale is used (0, not at all; 3 almost every day). Participants responded to the increase in the frequency of experiencing difficulties in each area over the past 2 weeks. Previous work has demonstrated good psychometric characteristics (Abe et al., 2020); in the present study, the internal consistency was acceptable (α = 0.74).

##### Insomnia

The Insomnia Severity Index (ISI) (Smith and Trinder, 2001) was used. Participants responded on a 5-point scale ranging from 1 (not at all) to 5 (almost every day). Higher scores indicate greater severity of insomnia. Previous studies using the ISI supported its psychometric properties (Yu, 2010), and it has showed good internal consistency (α = 0.94) in this study.

### Statistical Analysis

The data for this study were based on self-reports from frontline medical staff, it was possible that there could be an issue with common method variance. We used Harman’s one-factor test to detect the presence of common method bias (Podsakoff et al., 2003). Results showed that there were nine factors with the eigenvalue greater than 1, and the first factor to explain the variance accounted for 27.48%, which was less than the critical value of 40%, indicating that the common method deviation was not obvious.

First, mean, standard deviation, and frequency of the socio-demographic characteristics of the sample were examined. We calculated prevalence estimates and frequency of the mental health symptoms among frontline medical staff during the COVID-19 epidemic. Second, for the main analysis, the descriptive information and correlation matrix were conducted. Third, after all the data were standardized, based on 5,000 bootstrap samples, the PROCESS macro (Hayes, 2013) was used to test the mediating effect of perceived stress between occupational stressors and three mental health symptoms respectively (model 4). Based on the established mediation model, we further tested the moderating role of social support between the association of occupational stressors, perceived stress, and three mental health symptoms respectively (model 15). The control variables were sex, age, education level, marital status, and length of service. The effects are significant when the confidence intervals exclude zero.

## Results

### Prevalence and Characteristics of Mental Health Symptoms

Table 2 shows the prevalence of three types of mental health problems in frontline medical staff during the COVID-19 epidemic. Overall, out of 525 frontline medical staff, 209 (39.8%) subjects had symptoms of anxiety and 50 (9.5%) had moderate and above moderate level of anxiety on the GAD-7; 157 (29.9%) subjects had symptoms of depression and 28 (5.3%) had moderate and above moderate level of depression on the PHQ-9; 199 (37.9%) subjects had symptoms of insomnia and 46 (8.8%) had moderate and above moderate level of insomnia on the ISI.

**TABLE 2:** The prevalence of mental health symptoms in frontline medical staff.

Mental health symptoms	participants (*n* = 525)
Anxiety	
No anxiety	316 (60.2%)
Mild anxiety	159 (30.3%)
Moderate anxiety	33 (6.3%)
Severe anxiety	17 (3.2%)
Depression	
No depression	368 (70.1%)
Mild depression	129 (24.6%)
Moderate depression	22 (4.2%)
Moderately severe or severe depression	6 (1.1%)
Insomnia	
Absence of insomnia	326 (62.1%)
Mild insomnia	153 (29.1%)
Moderate insomnia	40 (7.6%)
Severe insomnia	6 (1.1%)

*Data are n (%); percentages represent the distribution of variable categories among all participants.*

### Preliminary Analyses

Confirmatory factor analyses were performed to discern whether the anxiety, depression, and insomnia represented the symptoms of mental health had a good fit. A three-factor model, χ^2^ = 1013.07, *df* = 227, *p* < 0.001, comparative fit index (CFI) = 0.90, and root mean square error of approximation (RMSEA) = 0.08, in which anxiety and depression and insomnia were distinct constructs, fit the data better than a one-factor model (χ^2^ = 3243.17, *df* = 230, *p* < 0.001, CFI = 0.63, and RMSEA = 0.16). The result of these confirmatory factor analyses indicated the appropriateness of treating anxiety, depression, and insomnia as empirically distinct constructs.

Descriptive statistics and correlations for some variables measured are reported in Table 3. As expected, occupational stressors and perceived stress were positively associated with three types of mental health symptoms respectively (*ps* < 0.05). Social support was significantly and inversely correlated with anxiety, depression, and insomnia respectively (*ps* < 0.001). Social support was negatively associated with perceived stress (*p* < 0.001), but not with occupational stressors. In addition, in demographic variables, age was positively associated with occupational stressors and anxiety respectively (*ps* < 0.05); length of service was positively associated with occupational stressors and anxiety respectively (*ps* < 0.05); education level was positively associated with occupational stressors and depression respectively (*ps* < 0.05); marital status was significantly and inversely correlated with occupational stressors and anxiety respectively (*ps* < 0.05); and sex identification did not significantly correlate with any of the variables (*ps* > 0.05), as such it was excluded from the remaining analysis.

**TABLE 3:** Means, standard deviations, and correlations among study variables.

Variable	*M*	*SD*	1	2	3	4	5	6	7	8
1. Age	35.29	7.05	1							
2. Length of service	12.32	8.66	0.83***	1						
3. Occupational stressors	11.04	1.65	0.11*	0.11*	1					
4. Perceived stress	23.79	5.04	-0.06	-0.05	0.12**	1				
5. Social support	19.11	4.33	-0.003	0.008	0.02	-0.27***	1			
6. Anxiety	4.30	4.17	0.13**	0.11*	0.30***	0.46***	-0.18***	1		
7. Depression	3.36	3.67	-0.04	-0.06	0.24***	0.52***	-0.26***	0.71***	1	
8. Insomnia	6.47	5.47	-0.03	-0.08	0.09*	0.36***	-0.22***	0.47***	0.58***	1

Covariate: sex, age, education level, marital status, and length of service. *p < 0.05; ** p < 0.01; and *** p < 0.001.

### Main Effects Results

The variable occupational stressors were significantly associated with mental health symptoms respectively (anxiety: β = 0.73, *t* = 6.87, *p* < 0.001, Δ*R*^2^ = 0.08; depression: β = 0.55, *t* = 5.77, *p* < 0.001, Δ*R*^2^ = 0.06; and insomnia: β = 0.29, *t* = 1.99, *p* < 0.05, Δ*R*^2^ = 0.0037), providing support for Hypothesis 1 a/b/c. Occupational stressors were also significantly positively associated with perceived stress (β = 0.37, *t* = 2.76, *p* < 0.01, Δ*R*^2^ = 0.014). Perceived stress was significantly associated with mental health symptoms (anxiety: β = 0.39, *t* = 12.28, *p* < 0.001, Δ*R*^2^ = 0.22; depression: β = 0.38, *t* = 13.85, *p* < 0.001, Δ*R*^2^ = 0.22; and insomnia: β = 0.38, *t* = 8.71, *p* < 0.001, Δ*R*^2^ = 0.12).

### The Mediating Effects of Perceived Stress

The PROCESS macro (model 4) (Hayes, 2013) was used to test the mediating effect of perceived stress between occupational stressors and mental health symptoms, respectively. Significance of indirect effect was determined via 5,000 bias-corrected bootstrap confidence intervals using 1,000 bootstrap samples and 95% confidence intervals. The 95% confidence intervals that do not contain zero indicate effects that are significant.

**FIGURE 2:**
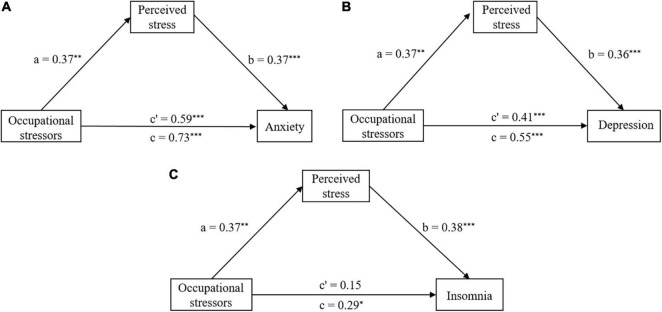
The mediation model of perceived stress, **(A)** Perceived stress mediation model for anxiety, **(B)** Perceived stress mediation model for depression, and **(C)** Perceived stress mediation model for insomnia. *p < 0.05; ^**^p < 0.01; and ^***^p < 0.001.

In the model of anxiety, as shown in Figure 2A, occupational stressors were significantly associated with anxiety indirectly through perceived stress [Effect = 0.13, SE = 0.05, 95% CI (0.04, 0.25)]. The direct effect of occupational stressors on anxiety was also significant [Effect = 0.59, SE = 0.09, 95% CI (0.41, 0.78)], confirming perceived stress as a partial mediator of the link between occupational stressors and anxiety.

In the model of depression, as shown in Figure 2B, occupational stressors were significantly associated with depression indirectly [Effect = 0.13, SE = 0.05, 95% CI (0.03, 0.25)], the direct effect of occupational stressors on depression was also significant [Effect = 0.41, SE = 0.08, 95% CI (0.25, 0.58)]. Perceived stress was again a partial mediator of the link between occupational stressors and depression.

In the model of insomnia, as shown in Figure 2C, the indirect association between occupational stressors and insomnia was significant [Effect = 0.14, SE = 0.06, 95% CI (0.04, 0.26)], but the direct effect of occupational stressors on insomnia was not significant [Effect = 0.15, SE = 0.14, 95% CI (−0.12, 0.42)]. These findings confirmed that perceived stress was a full mediator of the link between occupational stressors and insomnia.

In sum, the results supported Hypothesis 2. Specifically, perceived stress was a partial mediator of the link between occupational stressors and anxiety, depression symptoms, and was a full mediator of the link between occupational stressors and insomnia symptoms.

### The Moderating Effects of Social Support

Hypothesis 3 proposes that participants’ social support moderates the relationship between occupational stressors, perceived stress, and three mental health symptoms. Model 15 of Hayes’s (2013) PROCESS macro using 5,000 bias-corrected bootstrapped samples was used to test this relationship.

#### Anxiety

In the model of anxiety, as shown in Table 4, the interaction between occupational stressors and social support on anxiety was not significant (β = −0.02, *t* = −0.72, *p* = 0.47, Δ*R*^2^ = 0.0007), but the interaction between perceived stress and social support on anxiety was significant (β = −0.01, *t* = −2.08, *p* = 0.04, Δ*R*^2^ = 0.01) after accounting for the mediating role of perceived stress. Thus, social support moderates the indirect effect of occupational stressors on anxiety via perceived stress but does not moderate the direct effect of occupational stressors on anxiety.

**TABLE 4:** Testing the moderated mediation effect in the model of anxiety.

	Model a (Anxiety)		Model b (Anxiety)		Model c (Anxiety)	
	*b*	*T*	*b*	*t*	*b*	*t*
Occupational stressors	0.77	7.34***	0.62	6.48***	0.61	6.33***
Social support	−0.18	−4.55***	−0.07	−1.81	−0.06	−1.70
Occupational stressors × Social support	−0.05	−2.01*	−0.03	−1.28	−0.02	−0.72
Perceived stress			0.35	10.84***	0.34	10.59***
Perceived stress × Social support					-0.01	-2.08*
*R* ^2^	0.38		0.55		0.56	
*F*	12.77***		28.40***		25.88***	

*p < 0.05; **p < 0.01; ***p < 0.001.

**FIGURE 3:**
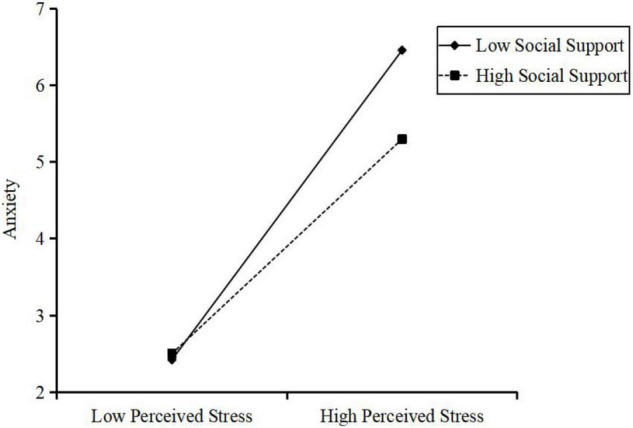
Interaction effect of social support and perceived stress on the anxiety symptom. High and low levels of perceived stress and social support represent one standard deviation above and below the mean, respectively.

To clarity the direction of the interaction, the variable anxiety was plotted on perceived stress, separately at high (1 SD above the mean) and low (1 SD below the mean) levels of social support. Figure 3 shows that low social support was associated with high level of anxiety for those with high level of perceived stress. However, for those with high social support, the level of anxiety was relatively low even when the level of perceived stress was notably high.

Furthermore, simple slope tests showed that participants with low level of social support (1 SD below the mean) and higher perceived stress combined were associated with more anxiety symptom (*slope* = 0.40, SE = 0.04, *p* < 0.001). For participants with a high level of social support, the effect of perceived stress on anxiety symptom was also significant (*slope* = 0.28, SE = 0.05, *p* < 0.001) but not as strong as that for participants with low social support.

Moreover, the bootstrap procedure confirmed that the indirect effect of occupational stressors on anxiety symptom through perceived stress was moderated by social support, because the index of moderated mediation was significant [β = −0.01, SE = 0.003, 95% CI = (−0.01, −0.0002)]. The indirect effect of perceived stress for participant with low social support [β = 0.15, SE = 0.06, 95% CI = (0.04, 0.27)] was stronger than those with high social support [β = 0.10, SE = 0.04, 95% CI = (0.02, 0.20)]. Therefore, our hypothesis 3a was supported.

#### Depression

In the model of depression, as shown in Table 5, the interaction between occupational stressors and social support on depression was not significant (β = −0.03, *t* = −1.30, *p* = 0.19, Δ*R*^2^ = 0.002), but the interaction between perceived stress and social support on depression was significant (β = −0.03, *t* = −4.28, *p* < 0.001, Δ*R*^2^ = 0.02) after accounting for the mediating role of perceived stress. This indicates that social support can moderate the indirect effect of occupational stressors on depression via perceived stress, but cannot moderate the direct effect of occupational stressors on depression.

**TABLE 5:** Testing the moderated mediation effect in the model of depression.

	Model a (Depression)		Model b (Depression)		Model c (Depression)	
	*b*	*t*	*b*	*t*	*b*	*t*
Occupational stressors	0.60	6.59***	0.46	5.65***	0.44	5.40***
Social support	–0.23	−6.73***	–0.13	−3.96***	–0.12	−3.79***
Occupational stressors × Social support	–0.07	−3.06**	–0.05	−2.41**	–0.03	–1.30
Perceived stress			0.33	11.90***	0.31	11.56***
Perceived stress × Social support					–0.02	−4.28***
*R* ^2^	0.40		0.58		0.60	
*F*	13.70***		32.96***		32.31***	

**p < 0.01; ***p < 0.001.

**FIGURE 4:**
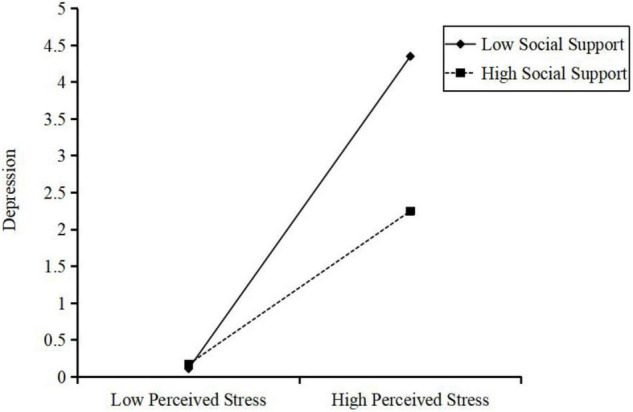
Interaction effect of social support and perceived stress on the depression symptom. High and low levels of perceived stress and social support represent one standard deviation above and below the mean, respectively.

To clarify the direction of the interaction, participants’ depression symptom was plotted on perceived stress, separately at high (1 SD above the mean) and low (1 SD below the mean) levels of social support. Figure 4 shows that low social support was associated with high level of depression for those with high level of perceived stress. However, for those with high social support, the level of depression was relatively low even with the high level of perceived stress.

Further, simple slope tests showed that low level of social support (1 SD below the mean), and higher perceived stress combined was associated with more depression symptom (*slope* = 0.42, SE = 0.04, *p* < 0.001). For participants with a high level of social support, the effect of perceived stress on depression symptom was also significant (*slope* = 0.21, SE = 0.04, *p* < 0.001) but not as strong as that for participants with low social support.

Moreover, the bootstrap procedure confirmed that the indirect effect of occupational stressors on depression symptom through perceived stress was moderated by social support, because the index of moderated mediation was significant [β = −0.01, SE = 0.004, 95% CI = (−0.02, −0.002)]. The indirect effect of perceived stress for participant with low social support [β = 0.16, SE = 0.06, 95% CI = (0.04, 0.28)] was stronger than those with high social support [β = 0.08, SE = 0.03, 95% CI = (0.02, 0.15)]. Therefore, our hypothesis 3b was supported.

#### Insomnia

In the model of insomnia, as shown in Table 6, the interaction between occupational stressors and social support on insomnia was not significant (β = −0.03, *t* = −0.80, *p* = 0.42, Δ*R*^2^ = 0.001), and the interaction between perceived stress and social support on insomnia was also not significant (β = −0.02, *t* = −1.85, *p* = 0.07, Δ*R*^2^ = 0.006). Thus, Hypothesis 3c was not supported.

**TABLE 6:** Testing the moderated mediation effect in the model of insomnia.

	Model a (Insomnia)		Model b (Insomnia)		Model c (Insomnia)	
	*b*	*t*	*b*	*t*	*b*	*t*
Occupational stressors	0.35	2.42*	0.20	1.47	0.18	1.33
Social support	–0.28	−5.21***	–0.17	−3.22**	–0.17	−3.13**
Occupational stressors × Social support	–0.06	–1.85	–0.04	–1.30	–0.03	–0.80
Perceived stress			0.33	7.27	0.32	7.05***
Perceived stress × Social support					–0.02	–1.85
*R* ^2^	0.29		0.41		0.42	
*F*	6.81***		13.17***		12.14***	

*p < 0.05; **p < 0.01; ***p < 0.001.

In sum, Hypothesis 3 was partially supported. Specifically, social support could moderate the indirect effect of occupational stressors on anxiety and depression, but not insomnia; social support cannot moderate the direct effect of occupational stressors on three mental health symptoms (anxiety, depression, and insomnia).

## Discussion

As an international public health emergency, COVID-19’s impact on mental health has gained widespread attention from the public. This study estimated the prevalence and severity of three mental health symptoms among frontline medical staff. It also evaluated the mediating role of perceived stress in the link between the occupational stressors and three mental health symptoms separately. Finally, it examined the moderating role of social support in the link among occupational stressors, perceived stress, and three mental health symptoms respectively.

### Prevalence and Characteristics of Mental Health Symptoms

The first finding of the study is that the prevalence of anxiety, depression and insomnia symptom among frontline medical staff were respectively 39.8, 29.9, and 37.9%. Although these incidence rates are within the estimated range of mental health symptom reported in the global studies of frontline medical staff (Luo et al., 2020; Sanghera et al., 2020), it is also noted that this result is inconsistent with findings of other studies (Lai et al., 2020; Tan et al., 2020). For instance, Tan et al. (2020) surveyed 470 health care workers in Singapore during the COVID-19 epidemic and found that the prevalence of anxiety, depression symptoms were 14.5 and 8.9%, respectively, which is much lower than the rate in this study. Another study reported that more than 70% of the respondents among 1,257 frontline health care workers experienced psychological distress, which is higher than the rate in this study (Lai et al., 2020). This difference could be related to various assessment scales used, different samples selected, and the different data analyses performed in these studies. In addition, the prevalence of mental health symptoms among general population in China was approximately 7.2–18.3% during the COVID-19 outbreak (Liang et al., 2020). Higher risk of infection and more occupational stressors may be the main reasons for the poorer mental health of frontline medical staff. The present study indicates that the substantial psychological impact of the pandemic on frontline medical staff, resulting in a range of mental health symptoms including anxiety, depression, and sleep disturbances. Thus, more attention should be paid to health professionals in future studies.

### Occupational Stressors and Three Mental Health Symptoms

The second finding of the study is that occupational stressors were positively associated with three mental health symptoms including anxiety, depression, and insomnia. These findings are in line with the first hypothesis and consistent with recent studies regarding the relationship between occupational stressors and mental distress (Sauerbrei and Pham, 1986; Pappa et al., 2020; Abbas et al., 2021). Extra workload can lead medical staff to work harder to combat the pandemic and it can also increase their risks for various mental health-related issues including anxiety, fear, depression, and discrimination (Abbas et al., 2021). This study highlights the importance of providing support for frontline medical staff to manage their occupational stressors in order to alleviate their mental health problems (Mo et al., 2020). It expands this view by comparing the effect of occupational stressors on anxiety, depression, and insomnia. Still, in contrast to anxiety (73%) and depression (55%), occupational stressors were found to account for only 29% of symptoms of insomnia. During the COVID-19 peak outbreak, stressful occupational circumstances may serve as an acute stressor, which can trigger more negative emotional symptoms than somatic symptoms. Under this situation, emotional acceptance-centric mindfulness-related techniques may be an essential learning tool for medical staff to help them manage occupational stressors in crisis-related situations alike a pandemic.

### The Mediating Effects of Perceived Stress

The third finding of the study is that perceived stress was a strong statistical mediator of the relationship between occupational stressors and three mental health symptoms among frontline medical staff. It supports the second hypothesis and is consistent with results of previous stress studies (Harvey et al., 2010). This mechanism sheds light on why occupational stressors can lead to negative health outcomes.

Importantly, we found that perceived stress was a partial mediator of the link between occupational stressors and anxiety and depression among frontline medical staff. The cognitive appraisal theory shows that psychological distress is not solely the results of stressful events, but is also determined, to a large extent, by individual cognitive evaluation (Balieiro et al., 2011). The mediating roles of cognitive appraisal on the links from occupational stressors to mental health problems among nurses were confirmed in a previous study (Zhang et al., 2021a). It is noted that perceived stress may be due to limited resources available for frontline medical staff during the COVID-19 pandemic. Such resources may be essential in high-intensity work (Man et al., 2020). Still, some medical staff will perceive more stress beyond the work itself. The high perceived stress can indeed lead to the high feeling of negative emotions (i.e., anxiety and depression) among frontline medical staff (Wang and Wang, 2019; Abbas et al., 2021).

Furthermore, perceived stress was a full mediator and not a partial mediator of the link between occupational stressors and insomnia among frontline medical staff. This result indicated that the direct effect of occupational stressors on insomnia is weak, and the occupational stressors influences the insomnia mostly through the mediation role of perceived stress. Thus, to the extent possible, sustainable strategies may be developed and implemented to reduce perceived stress among frontline medical staff. A recent randomized controlled trial (RCT) found that a mindfulness-based stress reduction program was effective in reducing perceived stress among breast cancer survivors in China (Zhang et al., 2017). Future interventions and applied research may consider training medical staff to better understand and manage stress in order to alleviate the sleep impact of occupational stressors in crisis-related circumstances (Zhang et al., 2021a).

### The Moderating Effect of Social Support

The fourth finding of the study is that social support did not moderate the direct effects of occupational stressors and three mental health symptoms, but moderate the indirect effects of occupational stressors on anxiety and depression (not insomnia) via perceived stress. The results revealed that higher perceived stress was associated with more anxious and depressive emotional reactions in individuals with low social support. This is consistent with the buffer theory of social support, stating that low social support may be one of the main reasons why individuals suffering from the heavy and acute occupational stressors tend to experience negative emotions such as anxiety and depression (Huang et al., 2020; Zhang et al., 2020a).

Moreover, this study extends this view in three ways. First, social support can provide a buffer for individuals’ negative emotional reactions when dealing with occupational stressors. This buffer is legitimately not evident for medical staff somatic symptoms such as insomnia: COVID-19 leads to realistically enormous physical constraints including intermittent or irregular sleep hygiene in medical staff and these can limitedly be alleviated through social support. In addition, even without the pandemic, the prevalence of sleep disturbances among medical staff is still higher than general population (Zhang et al., 2021a). Thus, we should explore other factors that affect sleep to help reduce the sleep difficulty of medical staff. Secondly, this study found that social support only moderated the path between perceived stress and anxiety and depression. One possible explanation is that social support may intervene between a threatening event and a stress response by attenuating the stress appraisal (Baqutayan, 2011; Rollock and Lui, 2016). Frontline medical staff with higher levels of social support may be more likely to believe that others would provide the necessary resources to solve the problem when they encounter stress. This may help them redefine the perception about the potential harm of a situation and prevent a situation from being appraised as highly stressful (Zhou et al., 2019). Thirdly, another interesting finding is that social support did not moderate the direct effects of occupational stressors and three mental health symptoms. The buffer theory of social support reveal that the positive impact of social support in reducing psychological distress only occurs with perceived stress or distress (Cohen and Wills, 1985; Wang et al., 2020). For instance, a study found that for breast cancer patients, the most painful groups benefited the most from social support, but those with less distress did not get much additional benefit from social support (Mallinckrodt et al., 2012). Occupational stressors are valid concerns about work intensity, work difficulty, etc. The specialized knowledge and advanced skill of medical staff had crucial roles in treating patients with COVID-19. In addition, frontline medical staff showed a great sense of conscientiousness and the spirit of self-sacrifice during the COVID-19 epidemic. Liu et al. (2020) shared that Chinese health care providers reported stronger identification to their professional duty since COVID-19. Previous studies have also shown that medical staff with high levels of professional identity might perceive their occupation positively, which may improve their ability to resist stress, thereby reducing their perception of stress (Zhang et al., 2021b). When occupational stressors are not perceived as threatening to one sense of self, the stress response may not be activated. Thus, the result suggests that social support under perceived stress is more effective for helping individuals alleviate mental health problems.

## Limitations and Implications

Several limitations in this study should be mentioned. First, the respondents were all from Wenzhou, Zhejiang Province, which is one of the worst COVID-19 affected cities in China except Hubei Province. Certainly, the findings pertain may not generalize to frontline medical staff in other regions of China. Second, our study was based on cross-sectional design and causal inferences was limited. Future studies may adopt a longitudinal research design to provide evidence for the causal assumptions reported in this study. Thirdly, this study did not explore the potential relationship between demographic variables (such as age, marriage, culture, etc.) and mental health symptoms. In the future research, we will focus on it. Finally, we used a self-designed questionnaire to measure occupational stressors, and the results of the study relied on self-reported data, which may be subjective to some extent. Future research should choose standardized questionnaires whenever possible and collect data from multiple informants (e.g., family, co-workers, and supervisors) or multiple methods (e.g., objective and subjective measures).

Despite the aforementioned limitations, our study has meaningful implications in medical practice. First, based on the findings of high prevalence of anxiety, depression, and insomnia among frontline medical staff during the COVID-19 epidemic, the Chinese hospital managers may attend more to the growing concern of mental health among medical staff by increasing mental health awareness training, establishing mental health risk assessment, and empowering efficient coping interventions. Second, there is a significant association between occupational stressors and mental health symptoms among the frontline medical staff. Continuing medical education and targeted training in biological disaster ability can reduce occupational stressors, and thus improve the mental health of the medical staff. Organizational training on adequate work-related climate to navigate crisis interventions may also accentuate structurally-driven occupational stressors. Third, perceived stress is an important mechanism by which the occupational stressors of unexpected public crisis affect mental health among frontline medical staff. At the organizational level, the provision of adequate facilities, supportive health climate, inclusive work culture, along with wellbeing incentives, and can help medical staff to reduce their perceived stress (Abbas et al., 2021). At the medical staff level, in addition to strengthening psychological resilience, appropriate coping styles that center on realistic and accurate interpretation of stressful circumstances should be attained to correctly handle stressful events. Furthermore, some COVID-19 medical staff with sever sleep disorders should encourage them to seek help from sleep specialists or receive prescribed medical treatment. Finally, social support is another essential component of the mental health of the frontline medical staff. A multifaceted social support program based on the Chinese cultural values may be developed to promote mental health among the medical staff (Zhang et al., 2020a). In the workplace, harmonious interpersonal relationship with colleagues and supervisors should be encouraged. The services and sacrifices of medical staff should also be recognized and appreciated by the civil society and the media.

## Data Availability Statement

The original contributions presented in the study are included in the article/supplementary material, further inquiries can be directed to the corresponding authors.

## Ethics Statement

The studies involving human participants were reviewed and approved by the Ethics Committee of the Wenzhou Medical University. The patients/participants provided their written informed consent to participate in this study. Written informed consent was obtained from the individual(s) for the publication of any potentially identifiable images or data included in this article.

## Author Contributions

DB, LC, YLi, YZ, YLu, and FZ participated in research design. DB, LC, XL, AN-A, KX, LH, HW, and ZC collected the data. DB, LC, YLi, YZ, YLu, and GS conducted the data analysis. DB, LC, YLi, YZ, YLu, FZ, XL, and AN-A wrote and contributed to the writing of the manuscript. All authors have read and agreed to the published version of the manuscript.

## Conflict of Interest

The authors declare that the research was conducted in the absence of any commercial or financial relationships that could be construed as a potential conflict of interest.

## Publisher’s Note

All claims expressed in this article are solely those of the authors and do not necessarily represent those of their affiliated organizations, or those of the publisher, the editors and the reviewers. Any product that may be evaluated in this article, or claim that may be made by its manufacturer, is not guaranteed or endorsed by the publisher.

## Footnotes

1
www.wjx.cn

## References

[B1] Abbas S. Al-Abrrow H. Abdullah H. O. Alnoor A. Khattak Z. Z. Khaw K. W. (2021). Encountering Covid-19 And Perceived Stress And The Role Of A Health Climate Among Medical Workers. *Curr. Psychol.* 23 1–14. 10.1007/s12144-021-01381-8 33519147 PMC7823189

[B2] Abe C. Denney D. Doyle A. Cullum M. Adams J. Perven G. (2020). Comparison Of Psychiatric Comorbidities And Impact On Quality Of Life In Patients With Epilepsy Or Psychogenic Nonepileptic Spells. *Epilepsy Behav.* 102:106649. 10.1016/j.yebeh.2019.106649 31759316

[B3] Allsopp K. Brewin C. R. Barrett A. Williams R. Hind D. Chitsabesan P. (2019). Responding To Mental Health Needs After Terror Attacks. *BMJ* 366:L4828. 10.1136/bmj.l4828 31409609

[B4] Balieiro M. C. Dos Santos M. A. Dos Santos J. E. Dressler W. W. (2011). Does Perceived Stress Mediate The Effect Of Cultural Consonance On Depression? *Transcult. Psychiatry* 48 519–538. 10.1177/1363461511418873 22021106

[B5] Baqutayan S. (2011). Stress And Social Support. *Indian J. Psychol. Med.* 33 29–34. 22021950 10.4103/0253-7176.85392 PMC3195151

[B6] Bizri M. Kassir G. Tamim H. Kobeissy F. Hayek S. E. (2021). Psychological Distress Experienced By Physicians And Nurses At A Tertiary Care Center In Lebanon During The Covid-19 Outbreak. *J. Health. Psychol.* 27, 1288–1300. 10.1177/1359105321991630 33567926 PMC7879044

[B7] Carpenter R. (2016). A Review Of Instruments On Cognitive Appraisal Of Stress. *Arch. Psychiatr. Nurs.* 30 271–279. 10.1016/j.apnu.2015.07.002 26992882

[B8] Chen W.-Q. Siu O.-L. Lu J.-F. Cooper C. L. Phillips D. R. (2009). Work Stress And Depression: The Direct And Moderating Effects Of Informal Social Support And Coping. *StressHealth* 25 431–443.

[B9] Cohen S. Wills T. A. (1985). Stress, Social Support, And The Buffering Hypothesis. *Psychol. Bull.* 98 310–357. 3901065

[B10] Galbraith N. Boyda D. Mcfeeters D. Hassan T. (2021). The Mental Health Of Doctors During The Covid-19 Pandemic. *Bjpsych. Bull.* 45 93–97. 10.1192/bjb.2020.44 32340645 PMC7322151

[B11] Greenslade J. H. Wallis M. Johnston A. N. B. Carlström E. Wilhelms D. B. Crilly J. (2020). Key Occupational Stressors In The Ed: An International Comparison. *Emerg. Med. J.* 37 106–111. 10.1136/emermed-2018-208390 31551289

[B12] Gustafsson H. Skoog T. (2012). The Mediational Role Of Perceived Stress In The Relation Between Optimism And Burnout In Competitive Athletes. *Anxiety Stress Coping* 25 183–199. 10.1080/10615806.2011.594045 21726158

[B13] Harvey A. Nathens A. B. Bandiera G. Leblanc V. R. (2010). Threat And Challenge: Cognitive Appraisal And Stress Responses In Simulated Trauma Resuscitations. *Med. Educ.* 44 587–594. 10.1111/j.1365-2923.2010.03634.x 20604855

[B14] Hayes A. (2013). *Introduction To Mediation, Moderation, And Conditional Process Analysis: A Regression-Based Approach.* New York: Guilford.

[B15] Huang Q. An Y. Li X. (2020). Coping Strategies As Mediators In The Relation Between Perceived Social Support And Job Burnout Among Chinese Firefighters. *J. Health Psychol.* 2020:1359105320953475. 10.1177/1359105320953475 32883114

[B16] Kang L. Li Y. Hu S. Chen M. Yang C. Yang B. X. (2020). The Mental Health Of Medical Workers In Wuhan, China Dealing With The 2019 Novel Coronavirus. *Lancet Psychiat.* 7:E14. 10.1016/S2215-0366(20)30047-X 32035030 PMC7129673

[B17] Kneavel M. (2021). Relationship Between Gender. *Psychol. Rep.* 124 1481–1501. 32635816 10.1177/0033294120939844

[B18] Kroenke K. Spitzer R. L. Williams J. B. (2001). The Phq-9: Validity Of A Brief Depression Severity Measure. *J. Gen. Intern. Med.* 16 606–613. 10.1046/j.1525-1497.2001.016009606.x 11556941 PMC1495268

[B19] Kshtriya S. Kobezak H. M. Popok P. Lawrence J. Lowe S. R. (2020). Social Support As A Mediator Of Occupational Stressors And Mental Health Outcomes In First Responders. *J. Community Psychol.* 48 2252–2263. 10.1002/jcop.22403 32841385

[B20] Lai J. Ma S. Wang Y. Cai Z. Hu J. Wei N. (2020). Factors Associated With Mental Health Outcomes Among Health Care Workers Exposed To Coronavirus Disease 2019. *JAMA Netw. Open* 3:E203976. 10.1001/jamanetworkopen.2020.3976 32202646 PMC7090843

[B21] Lakey B. Cronin A. (2008). “Chapter 17 - Low Social Support And Major Depression: Research, Theory And Methodological Issues,” in *Risk Factors In Depression*, eds Dobson K. S. Dozois D. J. A. (San Diego: Elsevier).

[B22] Leung D. Y. Lam T. H. Chan S. S. (2010). Three Versions Of Perceived Stress Scale: Validation In A Sample Of Chinese Cardiac Patients Who Smoke. *BMC Public Health* 10:513. 10.1186/1471-2458-10-513 20735860 PMC2939644

[B23] Li X. Lyu H. (2020). Epidemic Risk Perception. *Front. Psychol.* 11:563741. 10.3389/fpsyg.2020.563741 33643107 PMC7902491

[B24] Liang Y. Wu K. Zhou Y. Huang X. Zhou Y. Liu Z. (2020). Mental Health In Frontline Medical Workers During The 2019 Novel Coronavirus Disease Epidemic In China: A Comparison With The General Population. *Int. J. Environ. Res. Public Health* 17:6550. 10.3390/ijerph17186550 32916836 PMC7558595

[B25] Liu Q. Luo D. Haase J. E. Guo Q. Wang X. Q. Liu S. (2020). The Experiences Of Health-Care Providers During The Covid-19 Crisis In China: A Qualitative Study. *Lancet Glob. Health* 8 E790–E798. 10.1016/S2214-109X(20)30204-7 32573443 PMC7190296

[B26] Luo M. Guo L. Yu M. Jiang W. Wang H. (2020). The Psychological And Mental Impact Of Coronavirus Disease 2019 (Covid-19) On Medical Staff And General Public - A Systematic Review And Meta-Analysis. *Psychiat. Res.* 291:113190. 10.1016/j.psychres.2020.113190 32563745 PMC7276119

[B27] Ma H. Qiao H. Qu H. Wang H. Huang Y. Cheng H. (2020). Role stress, social support and occupational burnout among physicians in China: a path analysis approach. *Int. Health* 12 157–163. 10.1093/inthealth/ihz054 31343067 PMC11973427

[B28] Mallinckrodt B. Armer J. M. Heppner P. P. (2012). A Threshold Model Of Social Support. *J. Couns. Psychol.* 59 150–160. 10.1037/a0026549 22229798 PMC3354567

[B29] Man M. A. Toma C. Motoc N. S. Necrelescu O. L. Bondor C. I. Chis A. F. (2020). Disease Perception And Coping With Emotional Distress During Covid-19 Pandemic: A Survey Among Medical Staff. *Int. J. Environ. Res. Public Health* 17:4899. 10.3390/ijerph17134899 32645962 PMC7369835

[B30] Mark G. Smith A. P. (2012). Occupational Stress. *Br. J. Health Psychol.* 17 505–521. 22107162 10.1111/j.2044-8287.2011.02051.x

[B31] Mo Y. Deng L. Zhang L. Lang Q. Liao C. Wang N. (2020). Work Stress Among Chinese Nurses To Support Wuhan In Fighting Against Covid-19 Epidemic. *J. Nurs. Manag.* 28 1002–1009. 10.1111/jonm.13014 32255222 PMC7262235

[B32] Moreno Fortes A. Tian L. Huebner E. S. (2020). Occupational Stress And Employees Complete Mental Health: A Cross-Cultural Empirical Study. *Int. J. Environ. Res. Public Health* 17:3629. 10.3390/ijerph17103629 32455763 PMC7277686

[B33] Moseley R. L. Turner-Cobb J. M. Spahr C. M. Shields G. S. Slavich G. M. (2021). Lifetime And Perceived Stress, Social Support, Loneliness, And Health In Autistic Adults. *Health Psychol.* 40 556–568. 10.1037/hea0001108 34618502 PMC8513810

[B34] Okun M. A. Melichar J. F. Hill M. D. (1990). Negative Daily Events, Positive And Negative Social Ties, And Psychological Distress Among Older Adults. *Gerontologist* 30 193–199. 10.1093/geront/30.2.193 2347500

[B35] Pappa S. Ntella V. Giannakas T. Giannakoulis V. G. Papoutsi E. Katsaounou P. (2020). Prevalence Of Depression. *Brain Behav. Immun.* 88 901–907. 32437915 10.1016/j.bbi.2020.05.026 PMC7206431

[B36] Podsakoff P. M. Mackenzie S. B. Lee J. Y. Podsakoff N. P. (2003). Common method biases in behavioral research: a critical review of the literature and recommended remedies. *J. Appl. Psychol.* 88, 879–903. 10.1037/0021-9010.88.5.879 14516251

[B37] Praharso N. F. Tear M. J. Cruwys T. (2017). Stressful Life Transitions And Wellbeing: A Comparison Of The Stress Buffering Hypothesis And The Social Identity Model Of Identity Change. *Psychiat. Res.* 247 265–275. 10.1016/j.psychres.2016.11.039 27936438

[B38] Quick J. C. Henderson D. F. (2016). Occupational Stress: Preventing Suffering, Enhancing Wellbeing. *Int. J. Environ. Res. Public Health* 13:459. 10.3390/ijerph13050459 27136575 PMC4881084

[B39] Raffaelli M. Andrade F. C. D. Wiley A. R. Sanchez-Armass O. Edwards L. L. Aradillas-Garcia C. (2013). Stress, Social Support, And Depression: A Test Of The Stress-Buffering Hypothesis In A Mexican Sample. *J. Adolesc. Res.* 23 283–289.

[B40] Reskati M. H. Shafizad M. Aarabi M. Hedayatizadeh-Omran A. Khosravi S. Elyasi F. (2021). Mental Health Status And Psychosocial Issues During Nationwide Covid-19 Quarantine In Iran In 2020: A Cross-Sectional Study In Mazandaran Province. *Curr. Psychol.* 1–17. [Epub ahead of print] 10.1007/s12144-021-02011-z 34253946 PMC8263010

[B41] Rollock D. Lui P. P. (2016). Do Spouses Matter? Discrimination, Social Support, And Psychological Distress Among Asian Americans. *Cultur. Divers. Ethnic. Minor. Psychol.* 22 47–57. 10.1037/cdp0000045 25867552

[B42] Rusli B. N. Edimansyah B. A. Naing L. (2008). Working Conditions, Self-Perceived Stress, Anxiety, Depression And Quality Of Life: A Structural Equation Modelling Approach. *BMC Public Health* 8:48. 10.1186/1471-2458-8-48 18254966 PMC2267182

[B43] Sanghera J. Pattani N. Hashmi Y. Varley K. F. Cheruvu M. S. Bradley A. (2020). The Impact Of Sars-Cov-2 On The Mental Health Of Healthcare Workers In A Hospital Setting-A Systematic Review. *J. Occup. Health* 62:E12175. 10.1002/1348-9585.12175 33131192 PMC7603426

[B44] Sauerbrei E. E. Pham D. H. (1986). Placental Abruption And Subchorionic Hemorrhage In The First Half Of Pregnancy: Us Appearance And Clinical Outcome. *Radiology* 160 109–112. 10.1148/radiology.160.1.3520643 3520643

[B45] Smith S. Trinder J. (2001). Detecting Insomnia: Comparison Of Four Self-Report Measures Of Sleep In A Young Adult Population. *J. Sleep. Res.* 10 229–235. 10.1046/j.1365-2869.2001.00262.x 11696076

[B46] Spitzer R. L. Kroenke K. Williams J. B. Löwe B. (2006). A Brief Measure For Assessing Generalized Anxiety Disorder: The Gad-7. *Arch. Intern. Med.* 166 1092–1097. 10.1001/archinte.166.10.1092 16717171

[B47] Suleman Q. Hussain I. Shehzad S. Syed M. A. Raja S. A. (2018). Relationship Between Perceived Occupational Stress And Psychological Well-Being Among Secondary School Heads In Khyber Pakhtunkhwa. Pakistan. *PLoS One* 13:e0208143. 10.1371/journal.pone.0208143 30540807 PMC6291082

[B48] Tan B. Y. Q. Chew N. W. S. Lee G. K. H. Jing M. Goh Y. Yeo L. L. L. (2020). Psychological Impact Of The Covid-19 Pandemic On Health Care Workers In Singapore. *Ann. Intern. Med.* 173 317–320. 32251513 10.7326/M20-1083 PMC7143149

[B49] Walton M. Murray E. Christian M. D. (2020). Mental Health Care For Medical Staff And Affiliated Healthcare Workers During The Covid-19 Pandemic. *Eur. Heart J. Acute. Cardiovasc. Care* 9 241–247. 10.1177/2048872620922795 32342698 PMC7189614

[B50] Wang W. Wu X. Liu A. Lan X. (2020). Moderating Role Of Social Support In The Relationship Between Posttraumatic Stress Disorder And Antisocial Behavior In Adolescents After The Ya’an Earthquake. *Psych. J.* 9 350–358. 10.1002/pchj.343 31968397

[B51] Wang Y. Wang P. (2019). Perceived Stress And Psychological Distress Among Chinese Physicians: The Mediating Role Of Coping Style. *Medicine* 98:E15950. 10.1097/MD.0000000000015950 31169719 PMC6571215

[B52] Wang Z. Chen J. Boyd J. E. Zhang H. Jia X. Qiu J. (2011). Psychometric Properties Of The Chinese Version Of The Perceived Stress Scale In Policewomen. *PLoS One* 6:e28610. 10.1371/journal.pone.0028610 22164311 PMC3229602

[B53] Wang Z. Liu H. Yu H. Wu Y. Chang S. Wang L. (2017). Associations Between Occupational Stress, Burnout And Well-Being Among Manufacturing Workers: Mediating Roles Of Psychological Capital And Self-Esteem. *BMC Psychiat.* 17:364. 10.1186/s12888-017-1533-6 29141601 PMC5688661

[B54] WHO (2022). *Coronavirus Disease (Covid-19) Situation Dashboard.* Geneva: World Health Organization.

[B55] Williams D. R. (2018). Stress And The Mental Health Of Populations Of Color: Advancing Our Understanding Of Race-Related Stressors. *J. Health Soc. Behav.* 59 466–485. 10.1177/0022146518814251 30484715 PMC6532404

[B56] Xiao H. Zhang Y. Kong D. Li S. Yang N. (2020). The Effects Of Social Support On Sleep Quality Of Medical Staff Treating Patients With Coronavirus Disease 2019 (Covid-19) In January And February 2020 In China. *Med. Sci. Monit.* 26:E923549. 10.12659/MSM.923549 32132521 PMC7075079

[B57] Yin H.-B. Lee J. C. K. Zhang Z.-H. Jin Y.-L. (2013). Exploring The Relationship Among Teachers’ Emotional Intelligence, Emotional Labor Strategies And Teaching Satisfaction. *Teach. Teach. Educ.* 35 137–145. 10.1186/s12913-016-1423-5 27409075 PMC4943498

[B58] Yu D. S. (2010). Insomnia Severity Index: Psychometric Properties With Chinese Community-Dwelling Older People. *J. Adv. Nurs.* 66 2350–2359. 10.1111/j.1365-2648.2010.05394.x 20722803

[B59] Zaka A. Shamloo S. E. Fiorente P. Tafuri A. (2020). Covid-19 Pandemic As A Watershed Moment: A Call For Systematic Psychological Health Care For Frontline Medical Staff. *J. Health Psychol.* 25 883–887. 10.1177/1359105320925148 32370621

[B60] Zhang X. Zhao K. Zhang G. Feng R. Chen J. Xu D. (2020b). Occupational Stress And Mental Health: A Comparison Between Frontline Medical Staff And Non-Frontline Medical Staff During The 2019 Novel Coronavirus Disease Outbreak. *Front. Psychiatry* 11:555703. 10.3389/fpsyt.2020.555703 33424651 PMC7785830

[B61] Zhang H. Ye Z. Tang L. Zou P. Du C. Shao J. (2020a). Anxiety Symptoms And Burnout Among Chinese Medical Staff Of Intensive Care Unit: The Moderating Effect Of Social Support. *BMC Psychiat.* 20:197. 10.1186/s12888-020-02603-2 32357865 PMC7195710

[B62] Zhang C. Q. Zhang R. Lu Y. Liu H. Kong S. Baker J. S. (2021a). Occupational Stressors. *J. Contextual. Behav. Sci.* 19:64–71. 33520643 10.1016/j.jcbs.2020.12.004 PMC7834481

[B63] Zhang Y. D. Gao Y. Q. Tang Y. Li Y. H. (2021b). The Role Of Workplace Social Capital On The Relationship Between Perceived Stress And Professional Identity Among Clinical Nurses During The Covid-19 Outbreak. *JPN J. Nurs. Sci.* 18:E12376. 10.1111/jjns.12376 32896954

[B64] Zhang J. Y. Zhou Y. Q. Feng Z. W. Fan Y. N. Zeng G. C. Wei L. (2017). Randomized Controlled Trial Of Mindfulness-Based Stress Reduction (Mbsr) On Posttraumatic Growth Of Chinese Breast Cancer Survivors. *Psychol. Health Med.* 22 94–109. 10.1080/13548506.2016.1146405 26853191

[B65] Zhou E. Qiao Z. Cheng Y. Zhou J. Wang W. Zhao M. (2019). Factors Associated With Depression Among Hiv/Aids Children In China. *Int. J. Ment. Health Syst.* 13:10. 10.1186/s13033-019-0263-1 30828360 PMC6381654

